# Structural Elucidation of Irish Organic Farmed Salmon (*Salmo salar*) Polar Lipids with Antithrombotic Activities

**DOI:** 10.3390/md16060176

**Published:** 2018-05-23

**Authors:** Alexandros Tsoupras, Ronan Lordan, Martina Demuru, Katie Shiels, Sushanta Kumar Saha, Constantina Nasopoulou, Ioannis Zabetakis

**Affiliations:** 1 Department of Biological Sciences, University of Limerick, V94 T9PX Limerick, Ireland; Alexandros.Tsoupras@ul.ie (A.T.); Ronan.Lordan@ul.ie (R.L.); marti.demuru@hotmail.it (M.D.); Ioannis.Zabetakis@ul.ie (I.Z.); 2 Department of Life and Environmental Sciences, University of Cagliari, via Ospedale 72, 09124 Cagliari, Italy; 3 Shannon Applied Biotechnology Centre, Limerick Institute of Technology, Moylish Park, V94 E8YF Limerick, Ireland; KatieShiels@lit.ie (K.S.); Sushanta.Saha@lit.ie (S.K.S.); 4 Department of Food Science and Nutrition, School of the Environment, University of the Aegean, GR 81400 Myrina, Lemnos, Greece

**Keywords:** salmon, polar lipids, platelet aggregation, platelet-activating factor (PAF), thrombin, antithrombotic, LC-MS, GC-MS, phosphatidylcholine, phosphatidylethanolamine, polyunsaturated fatty acids (PUFA), docosahexaenoic acid (DHA)

## Abstract

While several marine polar lipids (PL) have exhibited cardioprotective properties through their effects on the platelet-activating factor (PAF) pathways, salmon PL have not been tested so far. In this study, the antithrombotic activities of salmon PL were assessed in human platelets and the structural characterisation of bioactive salmon PL was performed by GC-MS and LC-MS analyses. PL from fillets of Irish organic farmed salmon (*Salmo salar*) were extracted and separated into several lipid subclasses by thin-layer chromatography (TLC), while their fatty acid profile was fully characterised by GC-MS. Salmon total lipids (TL), total neutral lipids (TNL), total polar lipids (TPL), and each PL subclass obtained by TLC were further assessed for their in vitro effects towards PAF-induced and thrombin-induced platelet aggregation in human platelets. Salmon PL exhibited antithrombotic effects on human platelet aggregation, mostly through their strong inhibitory effects against the PAF pathway with IC_50_ values comparable to other marine PL, but with lower effects towards the thrombin pathway. PL fractions corresponding to phosphatidylcholine and phosphatidylethanolamine derivatives exhibited the most potent anti-PAF effects, while LC-MS analysis putatively elucidated their structure/function relationship. Several diacyl-PC/PE and alkyl-acyl-PC/PE species containing mostly docosahexaenoic acid at their *sn*-2 glycerol-backbone may be responsible for the bioactivity. The data presented suggests that salmon contains PL with strong antithrombotic bioactivities.

## 1. Introduction

Over the past few decades, extensive research has been conducted concerning the anti-inflammatory and cardioprotective properties of fish oils and marine products. In the majority of these studies, most of the anti-inflammatory activities of fish oils were attributed to the ω3 polyunsaturated fatty acids (PUFA) content, including the presence of eicosapentaenoic acid (EPA) and docosahexaenoic acid (DHA), which are abundant in marine products [1]. It has been proposed that diets with a 1:1 ratio of ω6/ω3 PUFA may reduce the risk of many of the chronic diseases that are highly prevalent in Western societies and developing countries, including cardiovascular diseases (CVD) [2].

However, it is now well-established that more complex mechanisms underlie the beneficial effects of fish and fish oil consumption and administration of marine products that go far beyond the ω3 PUFA/eicosanoids-related mechanisms [3]. Other lipid constituents are also present in fish and fish oils that have different metabolic effects after absorption with distinct biological activities [3].

Recent research has highlighted the well-documented and promising beneficial effects of marine polar lipids (PL) towards inflammation related disorders, through a plethora of beneficial bioactivities [3,4]. These beneficial effects are not only limited to their superior incorporation into cell membranes and plasma lipoproteins, including high-density lipoprotein (HDL), but also include the bioavailability of their ω3 fatty acids, and mainly to their reported anti-inflammatory and antithrombotic activities through mechanisms such as the inhibition of the platelet-activating factor (PAF) pathway and the modulation of PAF metabolism [3,4].

PAF is a potent inflammatory mediator implicated in several inflammatory manifestations such as atherosclerosis and CVD, glomerulosclerosis and renal disorders, cancer, HIV-infection comorbidities, etc. [3,5,6,7,8]. Even though specific PAF antagonists and synthetic molecules have exhibited promising results in such disorders, the most prominent beneficial effects have been found in PL derived from several foods [3,9,10], including marine products without any reported side effects thus far [3,10,11,12]. These marine PL exhibit anti-inflammatory and antithrombotic activities through both inhibiting PAF activities and modulating its metabolism towards homeostatic reduction of PAF levels [3,4,13]. Through these bioactivities, marine PL have exhibited potent anti-atherogenic and cardioprotective effects in vitro and in vivo [3,10,11,12,13,14,15,16,17,18].

Salmon PL have yet to be assessed for their effects towards the PAF pathway. Therefore, for the first time this study examines the putative anti-inflammatory, antithrombotic, and cardioprotective effects of salmon PL derived from Irish organic farmed salmon, by assessing their inhibitory effects towards the PAF pathway and the thrombin pathway of platelet aggregation in human platelet-rich plasma (hPRP). In addition, these lipids were further separated into PL subclasses and structurally characterised by GC-MS and LC-MS analysis, in order to elucidate the structure and function relationship of salmon PL with strong antithrombotic and cardioprotective properties.

## 2. Results

### 2.1. TL, TPL, and TNL Content of Salmon Fillet Samples

Total lipids (TL) of all salmon samples (*n* = 6) were extracted and separated into total neutral lipids (TNL) and total polar lipid (TPL). The obtained amounts of TL, TPL, and TNL (expressed as g of lipids per 100 g of fish tissue) are given in Table 1. Salmon is categorised as an oily fish species and thus data of TL levels in this fish are higher than other fish species, which is in accordance with the literature [3]. In addition, the majority of lipids extracted were neutral, since the neutral lipid fraction (TNL) contributes approximately 60–84% of the TL, whereas the TPL fraction contributes approximately to the 16–40% of the TL.

### 2.2. TLC Analysis of Irish Organic Farmed Salmon Fillets Total Polar Lipids

**Figure 1:**
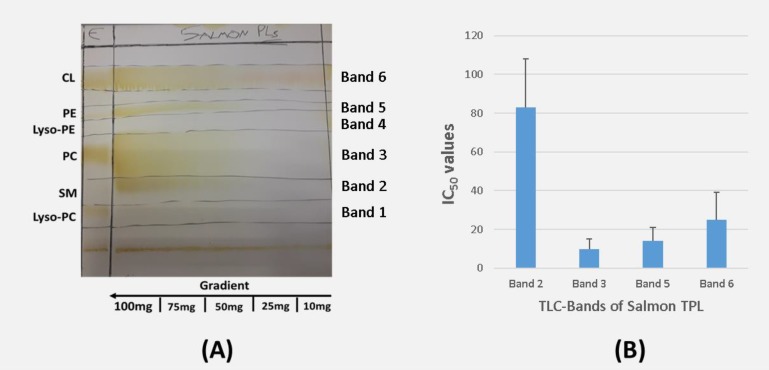
TLC analysis of the TPL of Irish organic farmed salmon fillets and the biological activity of each PL subclass towards PAF-induced platelet aggregation in hPRP. (**A**): Typical profile of the PL separation of salmon fillet on preparative TLC (Silica G). The elution system used for the separation of the TPL was chloroform:methanol:water 65:35:6 (*v*/*v*/*v*). PL: Polar lipids; E: mixture of standard PL from egg; Lyso-PC: lyso-phosphatidylcholine; SM: sphingomyelin; PC: phosphatidylcholine; Lyso-PE: lyso-phosphatidylethanolamine; PE: phosphatidylethanolamine; CL: cardiolipin; TLC; thin-layer chromatography. (**B**): Biological activities of the PL subclasses of the TLC bands towards PAF-induced platelet aggregation in hPRP. The results reflect the inhibitory strength of each lipid sample and are expressed as mean values of IC_50_ (μg of lipids in the aggregometer cuvette that causes 50% inhibition of PAF-induced aggregation of hPRP); The low IC_50_ value indicates strong inhibition of PAF-induced aggregation of hPRP; Lipid fractions of TLC bands 1 and 4 (corresponding to Lyso-PC and Lyso-PE) did not exhibit these bioactivities. hPRP: human platelet-rich plasma; PAF: platelet-activating factor; PL: polar lipids; TPL: total polar lipids.

Salmon TPL were further separated into PL subclasses and fractions by preparative TLC analysis. It was found that within the salmon TPL extracts several phospholipid species exist as depicted in the TLC bands (Figure 1A). PL fractions 1–6 have similar R_f_ values to those of lyso-phosphatidylcholine (L-PC), sphingomyelin (SM), phosphatidylcholine (PC), lyso-phosphatidylethanolamine (L-PE), phosphatidylethanolamine (PE), and cardiolipin (CL) respectively (Figure 1A).

### 2.3. Inhibitory Effect of Irish Organic Farmed Salmon Fillets Polar Lipids Towards Aggregation of Human Platelet-Rich Plasma (hPRP)

**Figure 2:**
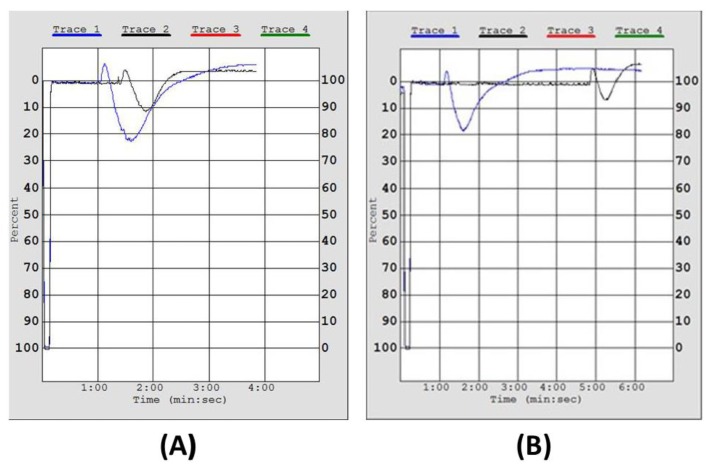
A representative chart of the 50% inhibitory effect of Irish organic farmed salmon fillet PL towards PAF-induced platelet aggregation in hPRP. Trace 1 (blue) in both A and B demonstrate the maximum-reversible aggregation of hPRP induced by 2.6 × 10^−8^ M of PAF; Trace 2 (black) demonstrates the effect of preincubation of hPRP with the appropriate concentration (IC_50_ value) of salmon TPL (**A**) and the corresponding TLC Band of PC (**B**), which causes 50% inhibition of PAF-induced hPRP aggregation. PAF: platelet-activating factor; hPRP: human platelet-rich plasma; TPL: total polar lipids; PC: phosphatidylcholine.

The in vitro study demonstrates that all salmon TPL extracts exhibited potent inhibition of PAF-induced aggregation of hPRP at very low doses, while much higher doses of these lipids were needed to inhibit the thrombin-induced aggregation of hPRP (Table 1). A representative chart for the in vitro effect of salmon TPL towards the PAF pathway of platelet aggregation of hPRP is depicted in Figure 2.

IC_50_ values reflect the inhibitory strength of each salmon TPL sample, because a low IC_50_ value indicates stronger inhibition of PAF-induced/thrombin-induced aggregation for a given salmon TPL concentration. Salmon TPL exhibited much higher inhibitory effects towards the PAF pathway than that of TL and TNL (Table 1). Thus, the intermediate IC_50_ value of the anti-inflammatory effect of the salmon TL can be attributed to the presence of both salmon TPL (with low IC_50_ values and thus higher inhibitory effects against PAF) and the salmon TNL (with higher IC_50_ value and thus lower inhibitory effects against PAF) within the TL fraction.

The mean IC_50_ value of the inhibitory effect of all the salmon TPL extracts towards PAF-induced aggregation was 45 ± 22 μg of TPL in the aggregometer cuvette (Table 1). This IC_50_ value is comparable with relative IC_50_ values of bioactive PL extracts from other fish species, but also from other food samples, which exhibited similar anti-inflammatory effects towards PAF-induced activation and aggregation of platelets [3,4,13,16,17,18,19,20,21,22]. Similarly, the TPL fraction exhibited the strongest inhibitory effect towards the thrombin pathway of platelet aggregation in hPRP, while higher amounts of both TL and TNL fractions were needed for such inhibition (Table 1). Thus, the intermediate IC_50_ value of the antithrombotic effect of the salmon TL can be similarly attributed to the presence of both salmon TPL (with low IC_50_ values and thus higher inhibitory effects against thrombin) and the salmon TNL (with higher IC_50_ value and thus lower inhibitory effects against thrombin) within the TL fraction. The mean IC_50_ value of the inhibitory effect of all the salmon TPL extracts towards thrombin-induced aggregation was 382 ± 39 μg (Table 1).

Nevertheless, the anti-thrombin effect of all salmon lipid samples tested (TL, TPL, and TNL respectively) were much lower than that of their effect on the PAF-induced platelet aggregation, as depicted by their much higher IC_50_ values against thrombin-induced platelet aggregation. This difference is much more intense in the TPL fraction where the IC_50_ values against thrombin are approximately one order of magnitude higher than that against PAF, implying that the in vitro antithrombotic effects of salmon PL mostly occur through their potent inhibitory effect on the PAF pathway of platelet aggregation in hPRP.

Furthermore, the PL subclasses in each of the TLC bands, which were obtained by the TLC separation of the TPL, were also tested for their ability to inhibit PAF-induced platelet aggregation of hPRP. The IC_50_ values of the bioactive lipid fractions in each TLC band are shown in Figure 1B. The TPL extract of salmon exhibited inhibitory properties, that are attributed to almost all the polar lipid fractions (Bands 2, 3, 5, and 6) apart from lipid fractions 1 and 4 that did not exhibit such an effect (Figure 1B). None of the fractions exhibited had any aggregatory properties in hPRP. PL fractions 3 and 5, which have similar R_f_ values to those of PC and PE exhibited the most potent inhibitory effect towards PAF-induced aggregation of hPRP. These results are in accordance with previous reported IC_50_ values against PAF for these PL subclasses (PC and PE) in the same TLC bands obtained from the TLC analysis of TPL extracts from several other fish species [3,4,13,15,17].

### 2.4. GC-MS Analysis of Salmon Polar Lipids

In all salmon TPL samples, PUFA were the most abundant fatty acid class followed by saturated fatty acids (SFA) and monounsaturated fatty acids (MUFA) (Table 2). More specifically, salmon TPL contains high amounts of ω3 PUFA with the most abundant ω3 fatty acids being the EPA (20:5ω3) and the DHA (22:6ω3) (Table 2). The most abundant ω6 fatty acids in salmon TPL were arachidonic acid (ARA; 20:4ω6) and linoleic acid (LA; 18:2ω6). The most abundant MUFA was oleic acid (18:1 c9) and the most abundant SFA was palmitic acid (16:0) and stearic acid (18:0). Interestingly, the ω3 fatty acid content was significantly higher than that of ω6 fatty acids, thus the ratio of ω6/ω3 was found to be approximately 1/2.5 (Table 2), which differs positively from the suggested value of 1 for this ratio.

In relation to the GC-MS analysis of the PL subclasses of each TLC band, the most abundant fatty acids found in band 3 (PC) were the ω3 fatty acids EPA (20:5ω3) and DHA (22:6ω3) from the PUFA, palmitic acid (16:0) and stearic acid (18:0) from the SFA, and oleic acid (18:1 c9), and palmitoleic acid (16:1 c9) from the MUFA (Table 3). Notably, the ω3 fatty acid content of salmon PC was significantly higher than that of ω6 fatty acids (Table 3), and thus the relative ratio of ω6/ω3 in salmon PC was found to be approximately 1/5, which is much lower than the value of 1 for this ratio, but was higher in comparison to the relative ratio found in the TPL.

In addition, similarly to the salmon PC subclass, the most abundant fatty acids found in band 5 (PE) were the ω3 fatty acid EPA from the PUFA, palmitic acid (16:0) and stearic acid (18:0) from the SFA, and oleic acid (18:1 c9) and palmitoleic acid (16:1 c9) from the MUFA (Table 3). However, the ω3 fatty acid DHA was not detected in the PE subclass of salmon. The ω3 fatty acid content of salmon PE was also higher than that of its content in ω6 fatty acids (Table 3), however the subsequent relative ratio of ω6/ω3 for this PL subclass had a value of 1/1.5 that even though it was still lower than the value of 1/1 for this ratio, it was however higher than the relative ratio of both salmon TPL and PC.

### 2.5. LC-MS Analysis of Salmon Polar Lipids

**Figure 3:**
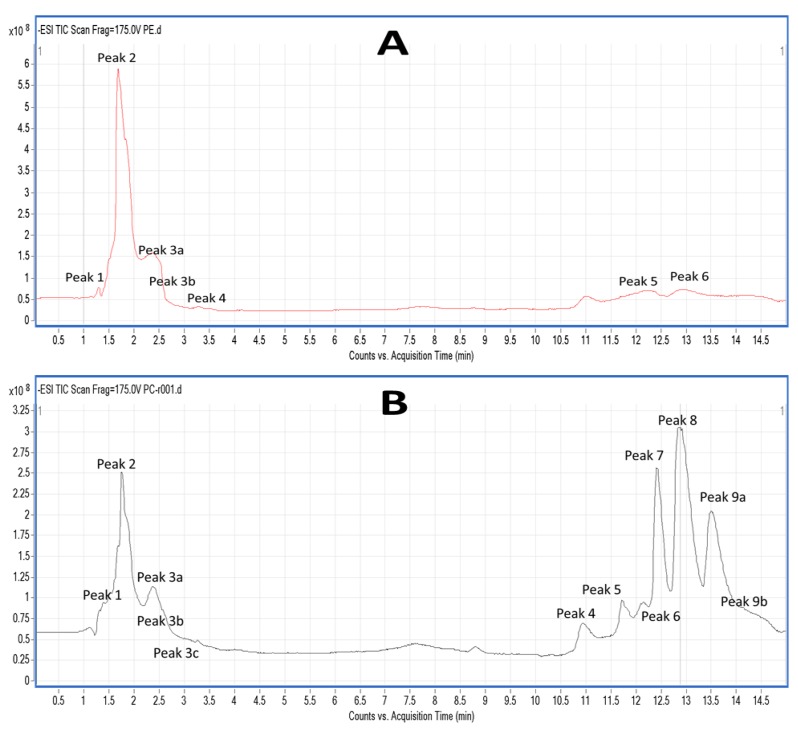
Representative HPLC chromatograms of salmon PL LC-MS analysis. (**A**) Depicts a representative chromatogram of the TLC fraction corresponding to salmon phosphatidylethanolamine (PE) derivatives, whereas (**B**) depicts a representative chromatogram of the TLC fraction corresponding to salmon phosphatidylcholine (PC) derivatives. Analysis was performed using a HPLC (Agilent 1260 series) equipped with Q-TOF mass spectrometer (Agilent 6520). The column used for the separations was an Agilent C18 Poroshell 120 column (2.7 µm, 3.0 × 150 mm). The composition of mobile phase (**A**) was 2 mM ammonium acetate in water and 2 mM ammonium acetate in 95% acetonitrile for mobile phase (**B**). Chromatographic separation was performed by gradient elution starting with 60% B for 1 min, then increasing to 90% B over 2.5 min. Subsequently, 90% B was held for 1.5 min and increased afterward to 100% over 5 min. Then, 100% B was held for 4 min, reducing afterward to 60% B over 0.5 min and hold for 1 min until the next run. The mobile phase flow rate was 0.3 mL/min until 5 min elapsed, increasing up to 0.6 mL/min until 10 min had elapsed and held at this flow until the end of the run. The injection volume was 10 μL.

Salmon TPL and the most bioactive TLC fractions against the PAF pathway, PC and PE, were further analysed by LC-MS. Characteristic chromatograms of the HPLC separation (by using a C18 reversed phase column) for both PE and PC TLC fractions are depicted in Figure 3. The HPLC analysis of PE revealed 6 major peaks (Figure 3A), while the HPLC analysis of PC revealed 9 major peaks with specific retention times (Figure 3B). The HPLC was equipped with a Q-TOF mass spectrometer, therefore MS analysis was also conducted simultaneously with the HPLC separation for each one of these peaks. Thus, specific MS spectra were obtained for each peak and several structures for these PC and PE moieties are proposed. Characterisation of these molecules was based on the acquired *m*/*z* values of the negative ions [M − H]^−^ for PE and the demethylated negative ions [M − CH_3_]^−^ for PC [23], and further verified by using the LIPID MAPS: Nature Lipidomics Gateway (www.lipidmaps.org), based on the lowest delta values during identification, in combination with their fatty acids contents that were acquired by both GC-MS and LC-MS analyses.

**Figure 4:**
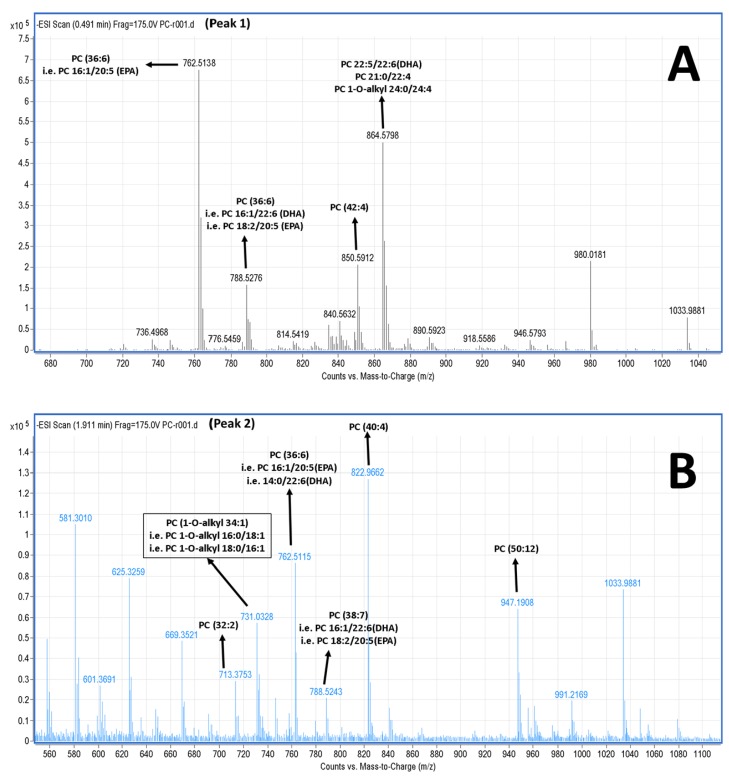

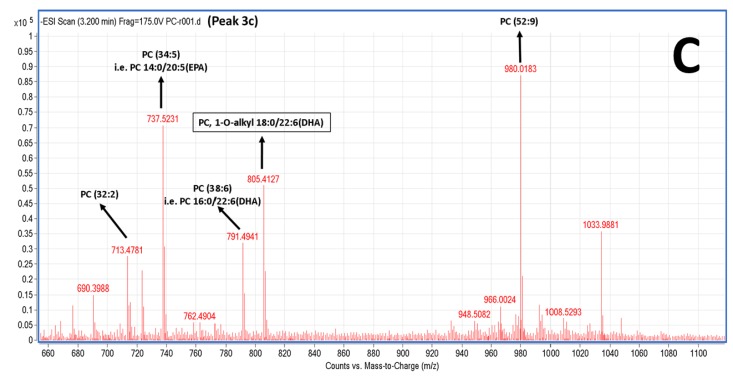
Representative mass spectra of PC species present in the relative TLC fraction of bioactive salmon PC. The LC-MS analysis demonstrated that the TLC fraction of the bioactive salmon PC contains a mixture of several PC species. (**A**–**C**) Mass spectra depicting the most abundant PC molecules eluted at peaks 1, 2, and 3 of the LC-MS analysis, respectively. The most probable and proposed identities of the PC species for each acquired *m*/*z* values were verified using the LIPID MAPS: Nature Lipidomics Gateway (www.lipidmaps.org), by using the lowest delta values during identification, in combination with their content of FA that was acquired by both GC-MS and LC-MS analyses. In all 3 peaks, several PC molecules are proposed baring either EPA or DHA in the *sn*-2 position of the glycerol backbone, whereas lower but considerable quantities of alkyl-acyl moieties also seem to be present in all spectra (**A**–**C**) that are outlined in squares. Interestingly, at peak 3c (elution time 3.2 min), a 1-*O*-alkyl (18:0)-2-*sn*-akyl (22:6, DHA)-3-glycerophosphocholine seems to be present in the TLC fraction of the bioactive salmon PC. FA: fatty acids; EPA: eicosapentaenoic acid; DHA: docosahexaenoic acid; PC: phosphatidylcholine.

The LC-MS analysis was carried out using a reverse phase column, by which the separation of the lipids is mostly based on the length of the non-polar acyl- or alkyl-groups in combination with their degree of unsaturation. Thus, PC and PE species baring PUFA were separated within small retention times (peaks 1–3), rather than other PC and PE species baring more saturated and longer chains within their structures that seem to be eluted in higher retention times (peaks 4–9). More specifically, in the negative ion mode, survey scans between 600 and 1000 *m*/*z* of MS1 (Figure 4) demonstrated a specific pattern of molecular species for PC eluted in peaks 1–3; it is proposed that many of these are diacyl-glycerol species containing either 14:0, 16:0, 16:1, 18:0, 18:1, or 18:2 fatty acids at the *sn*-1 position and mostly either the 22:6 fatty acid (DHA) or the 20:5 fatty acid (EPA) in the *sn*-2 position. Interestingly, 1-*O*-alkyl-2-*sn*-acyl-PC moieties also seem to be present in less, but considerable quantities, which are outlined in squares in Figure 4. Interestingly, at peak 3c (elution time 3.2 min), a specific 1-*O*-alkyl-(18:0)-2-*sn*-alkyl-(22:6,DHA)-3-PC with theoretical mass of 819.61 (with a relative demethylated negative ion [M − CH_3_]^−^ at 805.4 *m*/*z*) seems to be present in the TLC fraction of the bioactive PC of salmon PL. PC species eluted in peaks 4–9, with higher retention times, seem to have longer and more saturated carbon chains of their acyl-moieties. MS-data of these diacyl-PC species (with longer and more saturated fatty chains at both the *sn*-1 and *sn*-2 positions) are not shown, since such PC-species do not seem to possess any effect against platelet aggregation and have not been previously demonstrated to have similar bioactivity.

**Figure 5:**
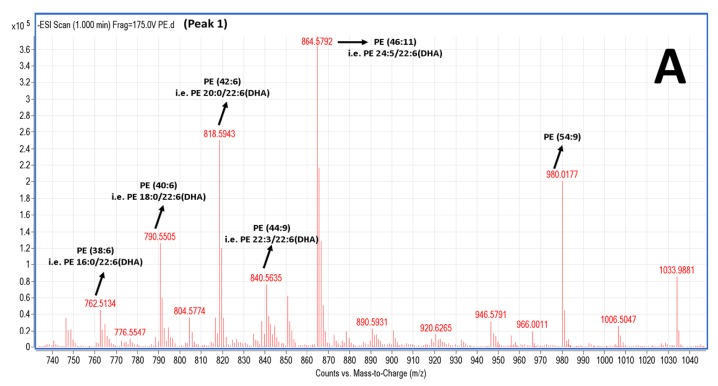

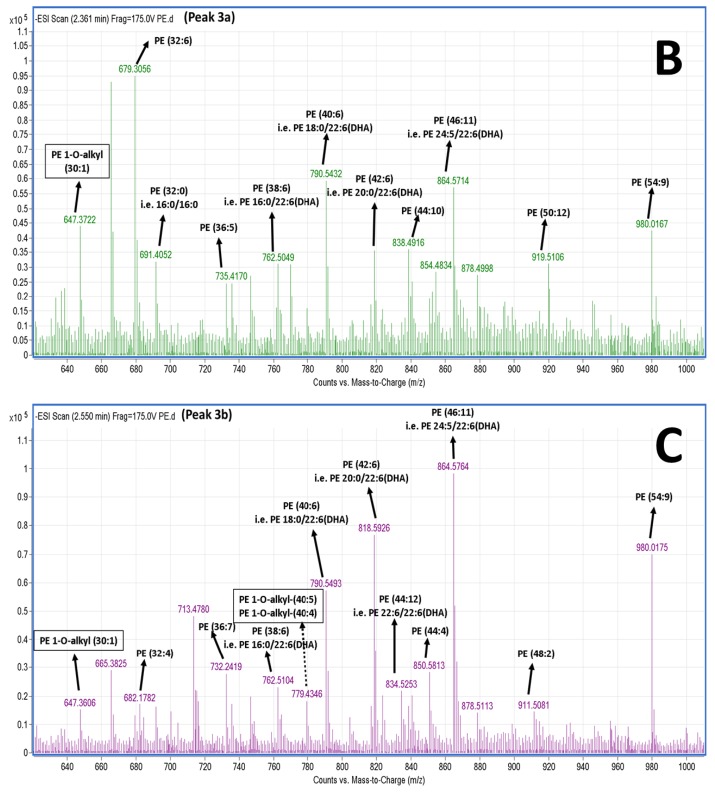
Representative mass spectra of PE species present in the relative TLC fraction of bioactive salmon PE. The LC-MS analysis showed that the TLC fraction of bioactive salmon PE contains a mixture of several PE species. (**A**–**C**) Mass spectra depicting the most abundant PE molecules eluted at peaks 1, 3a, and 3b of the LC-MS analysis, respectively. The most probable and proposed identities of PE species for each acquired *m*/*z* values were verified using the LIPID MAPS: Nature Lipidomics Gateway (www.lipidmaps.org), by using the lowest delta values during identification, in combination with their content in FA that was acquired by both GC-MS and LC-MS analyses. In all 3 peaks, several PE molecules are proposed with DHA in the *sn*-2 position of the glycerol backbone, whereas lower but considerable quantities of alkyl-acyl moieties seem to be present only at B and C spectra of the PE molecules eluted at peak 3, which are outlined in squares. FA: fatty acids; EPA: eicosapentaenoic acid; DHA: docosahexaenoic acid; PE: phosphatidylethanolamine.

In the negative ion mode, survey scans between 600 and 1000 *m*/*z* of MS1 (Figure 5) also exhibited a specific pattern of molecular species for PE; it is proposed that many of these are diacyl-glycerol species containing either 16:0, 18:0, 20:0 fatty acids at the *sn*-1 position and mostly the 22:6 (DHA) fatty acid in the *sn*-2 position. Interestingly, 1-*O*-alkyl-2-*sn*-acyl-PE moieties also seem to be present, yet in less considerable quantities that are outlined in squares in Figure 5. PE species eluted in peaks 5–6, with higher retention times, seem to have longer more saturated carbon chains of their acyl-moieties (data not shown).

## 3. Discussion

Marine oils have exhibited strong cardioprotective properties as has been extensively reviewed [24]. Thus, several dietary guidelines (i.e., The US Dietary Guidelines) and healthy dietary patterns (i.e., the Mediterranean diet) recommend fish intake at least twice weekly [3,11,25]. Intake of farmed Atlantic salmon twice weekly influences lipoprotein particle size, decreases serum concentrations of triglycerides, and increases HDL cholesterol in a manner associated with CVD risk reduction [26,27]. It has also been proposed that salmon may possess natural resistance mechanisms to reduce its own risk of atherosclerosis and cardiovascular risk, which corresponds well with its high investment in its own lipid metabolism and lipid profile [28]. In addition, oven baking salmon does not decrease the ω3 PUFA contents, indicating that baking salmon is an acceptable means of preparation that does not alter the potential health benefits of high ω3 seafood consumption [29]. Epidemiologic studies have shown that high dietary consumption of salmon and other oily fish, such as herring and trout, is associated with reduced rates of myocardial infarction, atherosclerosis, and other ischemic pathologies. In addition, dietary fish oil induces changes in cardiac function that might contribute to cardiovascular health benefits in humans and does so by modifying cardiac membranes within a dose range achievable in the human diet [30].

The therapeutic dose of ω3 fatty acids depend not only in the degree of severity of disease [2], but also in the form that these essential lipids are consumed with different digestion mechanisms and bioavailability in cell membranes and lipoproteins [3]. Furthermore, the results of very recent systematic reviews and meta-analyses have highlighted the lack of evidence to support the routine use of ω3 PUFA, especially when administered as triglycerides or esters, in the primary and secondary prevention of inflammatory disorders, since the beneficial effect of fish intake is likely to be mediated through the interplay of a wide range of nutrients abundant in fish [31,32,33,34,35]. It is now well-established that more complex mechanisms underlie the beneficial effects of fish/fish-oil consumption and administration of marine products that go far beyond the ω3 PUFA/eicosanoid-related mechanisms [3]. Other lipid constituents are also present in fish and fish oils that have different metabolic effects with distinct biological activities following absorption [3] and these lipid constituents are the focal point of the present manuscript.

Marine PL and especially marine phospholipids are gaining significant interest due to their plethora of beneficial bioactivities [3,11,12]. Dietary phospholipids have physiological functions that are different to dietary triglycerides. They can provide energy, they are a rich source of PUFA, and provide major components of cell membranes and plasma lipoproteins, thus they are important in nutrition and signal transduction for metabolic regulation, and the maintenance of living cells [3,36,37]. Marine phospholipids have a far superior incorporation into plasma lipoproteins including HDL and cell membranes following their consumption, but they may also provide a much higher bioavailability of their ω3 fatty acids, which are contained within their unique structures [3]. Additionally, marine PL have exhibited strong antithrombotic activities through specific mechanisms, including the potent inhibition of the PAF inflammatory pathway and the modulation of its metabolism towards homeostatic equilibration of PAF levels [3,4,11,12,13,16,17,18]. Through such bioactivities, marine PL have exhibited potent in vitro and in vivo anti-atherogenic and cardioprotective effects [3,4,11,12,13,16,17,18].

In the present study, it was found for the first time that Irish organic farmed salmon contain PL with potent antithrombotic effects against both the PAF pathway and thrombin pathway of human platelet aggregation. The in vitro anti-PAF effects of salmon PL are comparable with similar studies of PL from other fish species, such as sardines, sea bass, sea bream, cod, etc. [3,4,13,16,17,18,19]. However, although still very beneficial, the in vitro anti-thrombin effects of salmon PL were found to be much less potent (one order of magnitude lower) than their anti-PAF effects on human platelet aggregation, demonstrating that the TPL extracts of salmon contains PL that interact mostly via the PAF pathway.

In order to elucidate the relationship between the structure/activity of salmon PL, the salmon TPL extracts were separated by TLC analysis and all subclasses of PL in the TLC fractions obtained were further tested towards the PAF pathway, while further GC-MS and LC-MS analyses of the most bioactive PL subclasses were also conducted. It was found that specific lipid subclasses obtained by the TLC analysis of the salmon TPL, which correspond to PC and PE, exhibited the most potent inhibitory effect towards the PAF-induced human platelet aggregation, with respect to all the other lipid subclasses tested. These results are also in accordance with our groups previous studies of the equivalent TLC lipid subclasses of PC and PE in other fish species [15,16,17].

Since PAF is a potent inflammatory mediator that is implicated in several chronic diseases including atherosclerosis and CVD [7], renal disorders [38], cancer [5], HIV infection [39], etc., the novel findings of this study concerning the potent anti-PAF activities of salmon polar lipids seems to provide additional mechanistic evidence for the beneficial outcomes of the dietary intake of salmon and/or salmon oil in such disorders.

Concerning the fatty acid profile of these Irish organic farmed salmon PL, it was found that they contain high levels of ω3 PUFA, with the most abundant ω3 fatty acids being EPA and DHA. Furthermore, the ω3 fatty acid content was significantly higher than that of ω6 fatty acids, and thus the ratio of ω6/ω3 PUFA was found to be approximately 1/2.5. This fatty acid profile is comparable to previously reported salmon lipid profiles [40,41,42].

In addition, the fatty acid profile of the salmon PC and PE lipid subclasses, obtained by the TLC analysis of the salmon TPL, were also abundant in ω3 PUFA, with PC baring higher content of both EPA and DHA. The relative ratio of ω6/ω3 PUFA in these salmon PL was found to be approximately 1/5 and 1/1.5, respectively for salmon PC and PE.

Thus, the ratio of ω6/ω3 PUFA of salmon TPL, salmon PC, and salmon PE was found to be not only lower than the intermediate relative ratio of 1/1, but also much lower than that of Westernised diets, which is within the range of 5/1–20/1 [2]. It has been reported that high values of this ratio, which is observed mostly in Westernised diets are also correlated with a higher risk of chronic diseases [2,43]. Taking into account that marine PL possess high ability to incorporate in plasma lipoproteins and cell membranes and have high bioavailability of their ω3 PUFA [3], the much lower ratio of ω6/ω3 PUFA observed in salmon PL may also provide further evidence for their potential cardioprotective properties, since the lower this ratio is in the diet, the better health outcomes it provides against such chronic diseases [2].

Furthermore, by applying LC-MS analysis it is proposed that the PC species of the most bioactive TLC fractions of salmon PL (Figure 4) seem to contain both DHA and EPA at the *sn*-2 position, while the PE species seem to contain mostly DHA at the *sn*-2 position of their glycerol backbone (Figure 5). Such PL upon intake can be directly absorbed through the intestine as 20% of phospholipids from food intake are absorbed directly through intestine to blood and incorporated into blood lipoproteins [3]. Furthermore, phospholipids can be taken up/endocytosed by several cells with normal mechanisms, where upon phospholipase A_2_ (PLA_2_) activities allow the liberation of free DHA and EPA fatty acid from their *sn*-2 position, which can inhibit platelet activation with specific pathways but also through unknown mechanisms, especially for DHA [44]. Thus, these salmon PL that are rich in ω3 PUFA that are present mostly at the *sn*-2 position of the glycerol backbone, can serve as precursors (pro-drugs) of the ω3 PUFA that in their free fatty acid form have exhibited much higher anti-PAF and cardioprotective activities than their usually prescribed form as methyl esters [15].

In general, various PL in several foods have been found to exhibit either a weak agonistic (aggregatory) or an inhibitory effect on PAF-induced biological activities [3]. Some lipids, at low levels, could have a rather weak agonistic effect, but at higher levels their inhibitory activities prevail. Some other lipids might not show any agonistic activities but only inhibitory activities. The most classic PAF agonists are several alkyl-acyl-phospholipids [45]. In this study, LC-MS analysis revealed that considerable amounts of both alkyl-acyl-PC and alkyl-acyl-PE species are present in salmon PL. However, no aggregatory agonistic effect towards PAF was observed in all the PL tested. It seems that these molecules possess greater inhibitory effects against the PAF pathway than any possible agonistic effect from the constituent lipids in the PL fractions, and in this case, these components are practically acting mostly as PAF inhibitors.

Interestingly, at peak 3c (elution time 3.2 min) of the HPLC analysis of the PC subclasses, a specific 1-*O*-alkyl-(18:0)-2-*sn*-akyl-(22:6, DHA)-3-PC, with theoretical mass of 819.61 (with a relative demethylated negative ion [M − CH_3_]^−^ at 805,4 *m*/*z*) seems to be present in the PC TLC fraction of the salmon, which was bioactive against PAF. Taking into account that these fatty moieties seem to be present in this molecule and were also found within the most abundant molecules (18:0 fatty acid of the saturated ones and DHA (22:6) of the PUFA), by both GC-MS analyses (see Table 3) and LC-MS analyses of the salmon PL, this finding is in accordance with previously reported results of other bioactive lipid fractions in another fish species [15], where it was also found that the lipid composition of these bioactive fractions contained various glycerophospholipid species, where the majority of them have either 18:0 or 18:1 fatty acids in the *sn*-1 position and either 22:6 or 20:2 fatty acids in the *sn*-2 position.

It seems that the copresence of the DHA ω3 PUFA at the *sn*-2 position of such alkyl-acyl-phospholipids gives them higher inhibitory effects than any agonistic effect against PAF. Moreover, weak PAF agonists (with very low aggregatory activity; several orders of magnitude less than PAF) have been found to exhibit higher antiatherogenic activity than PAF inhibitors in vivo [13,46]. The presence of PL in PC and PE fractions that exhibit strong inhibitory effects against PAF-induced platelet aggregation is a potent indication that these salmon PL fractions contain biologically active compounds against PAF and consequently against atherogenesis and CVD. This means that unlike many other sources of lipids, Irish organic farmed salmon seem to be an excellent source of molecules with strong inhibitory effects against PAF.

Furthermore, salmon PC and in general marine PL baring ω3 PUFA and MUFA within their structures, have exhibited beneficial effects in several disorders through pleiotropic activities [3,37]. For example, salmon ω3 PUFA PC prevents the development of obesity related diseases through the suppression of lipogenic gene expression and the enhancement of lypolytic gene expression in the liver of obese rats [36]. In addition, salmon ω3 PUFA PC have also exhibited beneficial effects in the treatment of chronic liver diseases through its anti-inflammatory activities [47]. Administration of marine ω3 PUFA PC (i.e., PC from squid rich in DHA and PC from starfish rich in EPA) were shown to inhibit the growth of chemically induced colon cancer in vitro [48], while when administration of marine ω3 PUFA PC was combined with other marine phospholipids, the anti-cancer outcome in Caco-2 tumours was more potent [49]. Marine PL containing predominantly EPA and DHA were also shown to have remarkable lipid-lowering effects (reduction of total cholesterol, LDL, and triglyceride levels, while HDL increased significantly), while the supplementation of traditional fish oil (FA mostly bound to the triglyceride instead of PL) lowers only blood triglyceride levels and seems to have no effect on LDL and HDL levels [3,37]. It should be noted that most of these studies have been performed in vitro or in vivo and only limited evidence is available for the benefit of marine PL supplementation in humans [3,37], thus further research is required.

## 4. Materials and Methods

### 4.1. Materials and Instrumentation

All glass and plastic consumables, reagents, and solvents were of analytical grade and were purchased from Fisher Scientific Ltd. (Dublin, Ireland). 20 G safety needles and evacuated sodium citrate S-monovettes for blood sampling were purchased from Sarstedt Ltd. (Wexford, Ireland). The preparative TLC glass plates (20 × 20 cm) with silica gel G-60 of 1.0/2.0 mm thickness were purchased from Merck (Darmstadt, Germany). Bioassays on hPRP aggregations were carried out on a Chronolog-490 two channel turbidimetric platelet aggregometer (Havertown, PA, USA), coupled to the accompanying AGGRO/LINK software package. All platelet aggregation consumables were purchased from Labmedics LLP (Abingdon on Thames, UK). Standard PAF, thrombin and BSA were purchased from Sigma Aldrich (Wicklow, Ireland). Centrifugations were carried out on an Eppendorf 5702R centrifuge (Eppendorf Ltd., Stevenage, UK). Spectrophotometric analysis was carried out on a Shimadzu UV-1800 spectrophotometer (Kyoto, Japan). All GC-MS consumables were purchased from Apex Scientific Ltd. (Kildare, Ireland). Fatty acid methyl esters (FAME) standards for the GC-MS analysis and the egg phospholipid standard were purchased from Sigma Aldrich, Wicklow, Ireland. The GC-MS was a Varian 431-GC couples to a Varian 210-MS accompanied with the Varian Star Chromatography Workstation Version 6 software (Agilent Technologies, Palo Alto, CA, USA) and a NIST library (Gaithersburg, MD, USA).

### 4.2. Isolation of Salmon Total Lipids, Total Polar, and Total Neutral Lipids

Several (*n* = 6) 100 g samples of organic fresh farmed salmon fillets donated by Marine Harvest (private company), were homogenised and their total lipids (TL) were extracted into chloroform according to the method of Bligh and Dyer [50], flash vaporised under N_2_ stream, weighed, re-dissolved in 2 mL of CHCl_3_/MeOH 1:1 *v*/*v*, and stored at −20 °C.

One tenth of the TL was stored in sealed vials at −20 °C, while the rest was further separated into the total neutral lipids (TNL) fraction and the total polar lipids (TPL) fraction by the counter-current distribution method of Galanos and Kapoulas [51]. The acquired TNL and TPL fractions were weighed and stored under a nitrogen atmosphere at −20 °C for further analysis.

### 4.3. Fractionation of Salmon TPL by Thin-Layer Chromatography

The TLC analysis of the salmon TPL was performed as previously described for other fish species using an egg phospholipid standard (Sigma Aldrich, Wicklow, Ireland) [17]. Briefly, a gradient of approximately 10–100 mg of TPL was applied to the TLC plates. An elution system consisting of chloroform:methanol:water 65:35:6 (*v*/*v*/*v*), was utilised for the separation of TPL. Subsequently the plates were stained under iodine vapours. Six major bands appeared after the separation of the salmon TPL. Following the evaporation of the iodine vapours, the bands were scraped, and lipids were extracted from the silica gel according to the Bligh and Dyer method [50]. The chloroform phase was evaporated to dryness under nitrogen and lipids were weighed, re-dissolved in 1 mL of chloroform:methanol 1:1 (*v*/*v*), and stored at −20 °C in nitrogen until further analysis.

### 4.4. Human Platelet-Rich Plasma (hPRP) Aggregation Studies of Salmon Polar Lipids

The hPRP was separated as previously described [52] with some modifications. Briefly, for hPRP isolation, healthy human volunteers (*n* = 10) donated fasting blood samples. The Ethics Committee of the University of Limerick approved the protocol and it was performed in accordance with the Declaration of Helsinki. Healthy donors were fully aware that their blood samples were used in our study and written consent was provided to the specialised phlebotomist. A total of 50 mL of blood was collected from the median cubital vein or cephalic vein of each healthy volunteer via venepuncture using a 20 G safety needle, and blood was drawn into sodium citrate anticoagulant S-monovettes using the aspiration method (0.106 mol/L in a 1:10 ratio of citrate to blood; Sarstedt Ltd., Wexford, Ireland). The collected blood samples were centrifuged at 194 g for 18 min at 24 °C with no brake applied. The supernatant hPRP was then transferred to polypropylene tubes at room temperature for the aggregation bioassays, whereas platelet-poor plasma (PPP) was obtained by further centrifuging the specimens at 1465 g for 20 min 24 °C with no brake applied. hPRP was adjusted to 500,000 platelets/mL if required by addition of the respective volume of PPP according to the absorbance of the hPRP measured in the spectrophotometer. All procedures took place at 24 °C and all analyses were carried out within 2.5 h of the initial blood draw. PRP was stored at 24 °C before use in platelet aggregation bioassays.

Aliquots of standard PAF solution in chloroform/methanol (1:1 *v*/*v*) were evaporated under a stream of nitrogen and re-dissolved in BSA (2.5 mg BSA/mL saline) to obtain PAF solutions with final concentrations in the cuvette ranging from 2.6 × 10^−8^ to 2.6 × 10^−5^ mol/L. The examined salmon PL samples were also dissolved in BSA (2.5 mg BSA/mL saline). Standard active thrombin was dissolved in saline prior to testing. The ability of each selected sample to cause inhibition of either PAF-induced or thrombin-induced platelet aggregation was studied by adding various concentrations of each sample into the platelet suspension. Prior to testing, 250 µL of hPRP was added to an aggregometer cuvette at 37 °C with stirring at 1000 rpm and was calibrated using the PPP as a blank. The maximum-reversible PAF-induced/thrombin-induced platelet aggregation was determined as 100% aggregation, that was also used as baseline (0% inhibition), by adding PAF at approximately 2.6 × 10^−8^ M final concentration in the cuvette or thrombin at approximately 0.01–0.04 U/mL in the cuvette. The PAF-induced/thrombin-induced aggregation was calculated first at 0% inhibition of baseline in a cuvette, whereas after the preincubation of hPRP with the test samples in a variety of concentrations in a different cuvette, the same amount of PAF/thrombin was added and the reduced aggregation was calculated. Thus, a linear curve at the 20–80% range of the percentage of inhibition against PAF-induced/thrombin-induced aggregation of hPRP to the concentrations of each sample was deduced. From this curve, the concentration of the sample that led to 50% of PAF-induced/thrombin-induced aggregation of hPRP was calculated as the 50% inhibitory concentration value, also known as the IC_50_ value, for each sample. All experiments were performed in triplicate (*n* = 3), using a different donor’s blood sample for each replicate, to ensure reproducibility. The resulting IC_50_ values were expressed as a mean value of the mass of lipid (µg) in the cuvette ± standard deviation (SD).

### 4.5. GC-MS Analysis of Salmon Polar Lipids

Fatty acid methyl esters of salmon TPL and the PL subclasses of the PC and PE, which were obtained by the TLC analysis of salmon TPL, were prepared using a solution 0.5 N KOH in 90% CH_3_OH and extracted with n-hexane as previously described [53]. The fatty acid analysis was carried out using the internal standard method as previously described [22,53], with some modifications. In brief, a five-point calibration curve was prepared using five solutions of heptadecanoic acid methyl esters (17:0—50 ppm, 100 ppm, 200 ppm, 400 ppm, 800 ppm) and heneicosanoic acid (21:0—five 500 ppm injections) methyl esters standards. Five 1 µL injections of each solution were analysed with a Varian 410-Gas Chromatographer coupled to a Varian 210-MS detector equipped with a split/splitless injector (Agilent Technologies, Palo Alto, CA, USA). The ratio of the mean is of heptadecanoic acid (17:0) to that of the internal standard (21:0) and is used as the y-axis variable, whereas the concentration (ppm) of 17:0 is used as the x-axis variable of the calibration curve. The equation that described the curve was: y = 0.0041x + 0.12 with an R^2^ = 0.9969, where the ratio of the area of the analyte peak to that of the internal standard represents the y value for the above equation, subsequently the x-value represents the analyte concentration of a selected fatty acid in the lipid sample to be tested.

Separation of the FAME was achieved on an Agilent J&W DB-23 fused silica capillary column (60 m, 0.25 mm, i.d., 0.25 µm; Agilent, Santa Clara, CA, USA). The injector was set at 230 °C with a split ratio of 1:5; the injection volume was 1 µL. The carrier gas was high purity helium with a liner flow rate of 1 mL/min. The oven temperature was initially programmed to 100 °C for 5 min, raised to 240 °C at 3 °C/min, and finally isothermal at 240 °C for 10 min. FAME were identified using a pre-derivatised 37-component FAME standards mix (Sigma Aldrich, Wicklow, Ireland) by comparison of the retention times and their subsequent obtained MS spectra of the relative peaks with the aid of the Varian Star Chromatography Workstation Version 6 software (Agilent Technologies, Palo Alto, CA, USA) and a NIST library (Gaithersburg, MD, USA).

### 4.6. LC-MS Analysis of Salmon Polar Lipids

The salmon TPL and the most bioactive TLC fractions against the PAF pathway, PC and PE, were further analysed by LC-MS. Each of these lipid samples was separated in two half parts and dried in N_2_ stream. The first half part of each sample was saponified by adding 1.5 mL of saponification reagent, [2.5 M KOH: methanol (1:4, *v*/*v*)], which was gently vortexed. The vials were incubated at 72 °C for 15 min prior to addition of 225 µL of formic acid. Then, 1725 µL of chloroform and 375 µL of Milli-Q water was added, and vortexed to separate two layers. The chloroform layer containing free fatty acids was transferred carefully to amber vials and evaporated to dryness before storing at −20 °C until LC-MS analysis as per method described earlier [54].

Before LC-MS analysis, all the dried lipids were re-constituted in 500 µL of methanol: dichloromethane (2:1 *v*/*v*), centrifuged at 13,000 rpm for 6 min prior to filtering through 3 kDa ultra-centrifuge filters (Amicon Ultra 3k). Polar lipid and free fatty acid profiles were obtained in a HPLC (Agilent 1260 series) equipped with a Q-TOF mass spectrometer (Agilent 6520) and the source type was ESI. The column used for separations was an Agilent C18 Poroshell 120 column (2.7 µm, 3.0 × 150 mm). The composition of the mobile phase (A) was 2 mM ammonium acetate in water and 2 mM ammonium acetate in 95% acetonitrile for mobile phase (B). Chromatographic separation was performed by gradient elution starting with 60% B for 1 min, then increasing to 90% B over 2.5 min. Subsequently, 90% B was held for 1.5 min and increasing afterward to 100% over 5 min. Then, 100% B was held for 4 min, reducing afterward to 60% B over 0.5 min and hold for 1 min until the next run. The mobile phase flow rate was 0.3 mL/min until 5 min elapsed, increasing up to 0.6 mL/min after 10 min and held at this flow rate until the end of the run. The injection volume was 10 μL. The mass spectrometer was operated in negative ionization mode, scanning the lipids from *m*/*z* 50–1100. Drying gas flow rate, temperature, and nebuliser pressure were at 5 L min^−1^, 325 °C, and 30 psi, respectively. Fragmentor and skimmer voltages were kept at 175 V and 65 V, respectively, and the capillary voltage was 3500 V. In the negative ion mode, the monitoring reference masses used were 1033.988 and 112.9855, respectively. Assignment of free fatty acids and phospholipid species is based upon a combination of survey, daughter, precursor, and neutral loss scans. The identity of phospholipid species were verified using the LIPID MAPS: Nature Lipidomics Gateway (www.lipidmaps.org), by using the lowest delta values combined with the results obtained from the LC-MS analysis of FFA and the GC-MS analysis of FAME.

## 5. Conclusions

For the first time it has been demonstrated that salmon PL have the potential to inhibit platelet aggregation through their inhibitory effects on both the PAF and thrombin pathways. Their anti-PAF effects were found to be much higher than that of their anti-thrombin effects. These results emphasise the high quality of Irish organic farmed salmon fillets and their PL content with respect to protection from thrombosis. It is of interest to determine whether both the physiological and the beneficial effects of marine PL are the result of their polar head groups, the constituting PUFA (especially those incorporated at the *sn*-2 position), or both in combination. Since PUFA PL from other fish species also possess beneficial bioactivities [3,15], we believe that it is the coexistence of both these structural elements within the structures of marine PL that gives them the potential to exhibit pleiotropic beneficial effects in several chronic disorders [3,8]. Nevertheless, because of the extreme complexity in the aetiology of CVD and chronic inflammatory diseases, the best strategy could be to fully monitor the main features and effects of dietary patterns associated with a low prevalence and risk of such disorders. In such diets, including the Mediterranean diet, consumption of oily fish (e.g., sardines) rich in bioactive PL seems to be a major constituent and a key food. Salmon is a very worthy candidate to be incorporated into modern dietary patterns, as oily fish contain bioactive PL that prevent thrombosis.

Our current and future work aims to identify and elucidate the cardioprotective structures and mechanisms of fish PL that inhibit atherosclerosis, by applying a novel pro-drug/precursor approach [55] and by exploiting phospholipids containing ω3 PUFA within their structure, in order to reduce and inhibit the inflammatory and thrombotic PAF activity.

However, further ex vivo and in vivo studies are required in order to elucidate the structure/function relationship of salmon PL, their beneficial bioactivities, and their potential use as food supplements and nutraceuticals.

## Figures and Tables

**Table 1:** Content of TL, TPL, and TNL of Irish organic farmed salmon (*Salmo salar*) fillets and the inhibitory effect of these lipids towards the PAF/thrombin-pathway of platelet aggregation in hPRP.

	TL	TNL	TPL
Lipid Content * (g) ± SD	5.51 ± 1.90	4.66 ± 2.08	0.86 ± 0.36
IC_50_^†^ (µg) ± SD towards PAF inhibition	170 ± 32	432 ± 44	45 ± 22
IC_50_^†^ (µg) ± SD towards thrombin inhibition	451 ± 56	682 ± 62	382 ± 39

* Expressed as mean values of g of lipids per 100 g of salmon fillet (mean ± SD, *n* = 6); ^†^ IC_50_ values reflect the inhibitory strength of each lipid sample towards PAF-induced platelet aggregation in hPRP and is expressed as mean values of μg of lipids in the aggregometer cuvette that causes 50% of inhibition on PAF-induced platelets aggregation in hPRP ± standard deviation. TL: total lipids; TPL: total polar lipids; TNL: total neutral lipids; PAF: platelet-activating factor; hPRP; human platelet-rich plasma; SD: standard deviation.

**Table 2:** Fatty acid profile of the total polar lipids (TPL) of Irish organic farmed salmon fillets expressed as a percentage of total fatty acids (%FA) derived from the TPL and expressed as mg/kg of salmon fillet (mean ± SD, *n* = 3).

Fatty Acids	% FAs	mg/kg of Salmon
12:0	0.018 ± 0.001	1.3574 ± 0.096
14:0	2.107 ± 0.138	159.71 ± 15.33
14:1ω7 c9	0.098 ± 0.008	7.4086 ± 0.806
16:0	22.56 ± 0.658	1711.2 ± 95.30
16:1ω7 c9	1.992 ± 0.659	151.63 ± 55.74
18:0	6.331 ± 0.040	480.22 ± 38.98
18:1ω9 c9	13.31 ± 0.904	1009.8 ± 122.7
18:2ω6 c9, c12	6.938 ± 0.378	526.18 ± 50.54
18:2ω6 c11, c14	0.113 ± 0.008	8.5675 ± 0.275
18:3ω6 c6, c9, c12	0.047 ± 0.014	3.5902 ± 1.186
18:3ω3 c9, c12, c15	2.139 ± 0.123	161.78 ± 5.438
18:4ω3 c6, c9, c12, c15	0.695 ± 0.047	200.20 ± 257.0
20:0	0.157 ± 0.053	9.4081 ± 0.545
20:1ω9 c9	3.512 ± 0.208	265.62 ± 8.865
20:2ω6 c11, c14	0.890 ± 0.114	67.497 ± 10.30
20:3ω9 c5, c8, c11	0.771 ± 0.042	58.644 ± 7.951
20:4ω6 c5, c8, c11, c14	2.091 ± 0.120	158.41 ± 13.02
20:4ω3 c8, c11, c14, c17	0.347 ± 0.127	26.416 ± 10.45
20:5ω3 c5, c8, c11, c14, c17	10.02 ± 0.344	765.56 ± 37.49
22:1 c11	4.458 ± 0.590	339.03 ± 59.87
22:5ω6 c4, c7, c10, c13, c16	0.424 ± 0.116	32.176 ± 9.535
22:6ω3 c4, c7, c10, c13, c16, c19	12.94 ± 0.418	981.66 ± 92.63
Total SFA	32.54 ± 0.211	2425.5 ± 160.0
Total MUFA	24.08 ± 0.639	1835.3 ± 187.9
Total ω7 FA	2.448 ± 0.069	186.24 ± 58.80
Total ω9 FA	17.59 ± 0.927	1334.1 ± 187.9
Total PUFA	37.85 ± 0.527	3005.9 ± 366.4
Total ω3 PUFA	26.42 ± 0.419	2135.6 ± 317.2
Total ω6 PUFA	10.50 ± 0.131	796.43 ± 59.56
ω6/ω3		0.37 ± 0.08

TPL: total polar lipids; FA: fatty acids; SFA: saturated fatty acid; MUFA: monounsaturated fatty acid; PUFA: polyunsaturated fatty acid; ND: not detected.

**Table 3:** Fatty acid profile of the lipid fractions of PC and PE derived from salmon TPL, expressed as a percentage (%) of TPL fatty acids of salmon (mean ± SD, *n* = 3).

Fatty Acids	PC	PE
12:0	0.060 ± 0.004	0.034 ± 0.012
14:0	2.210 ± 0.212	5.424 ± 0.198
16:0	30.00 ± 0.488	20.76 ± 0.947
16:1ω7 c9	2.211 ± 0.121	1.883 ± 0.054
16:4ω3 c6, c9, c12, c15	0.290 ± 0.023	ND
18:0	8.171 ± 0.568	9.157 ± 0.543
18:1ω9 c9	7.937 ± 0.365	11.40 ± 0.044
18:1ω5 c13	2.369 ± 0.003	2.181 ± 0.269
18:2ω6 c9,c12	3.578 ± 0.068	2.298 ± 0.036
18:3ω3 c9, c12, c15	1.470 ± 0.033	1.562 ± 0.044
20:0	0.133 ± 0.029	0.945 ± 0.200
20:1ω9 c9	0.408 ± 0.011	1.054 ± 0.012
20:1ω7 c13	1.213 ± 0.097	6.670 ± 0.832
20:2ω6 c11, c14	0.516 ± 0.016	0.408 ± 0.001
20:3ω9 c5, c8, c11	0.252 ± 0.008	ND
20:4ω6 c5, c8, c11, c14	2.128 ± 0.139	1.012 ± 0.089
20:4ω3 c8, c11, c14, c17	0.785 ± 0.007	1.233 ± 0.030
20:5ω3 c5, c8, c11, c14, c17	9.289 ± 0.729	2.936 ± 0.123
22:1ω9 c11	2.203 ± 0.193	7.913 ± 0.716
22:5ω6 c4, c7, c10, c13, c16	2.553 ± 0.020	ND
22:6ω3 c4, c7, c10, c13, c16, c19	21.57 ± 1.407	ND
SFA	40.58 ± 1.301	36.32 ± 1.501
Total MUFA	16.34 ± 0.398	31.59 ± 0.247
Total ω7 FA	3.424 ± 0.218	2.937 ± 0.041
Total ω9 FA	10.80 ± 0.175	20.36 ± 0.747
Total PUFA	42.43 ± 2.172	9.449 ± 0.233
Total ω3 PUFA	33.41 ± 2.219	5.731 ± 0.109
Total ω6 PUFA	6.222 ± 0.054	3.718 ± 0.125
ω6/ω3	0.186 ± 0.075	0.649 ± 0.053

PC: phosphatidylcholine; PE: phosphatidylethanolamine; TPL: total polar lipids; FA: fatty acids; SFA: saturated fatty acid; MUFA: monounsaturated fatty acid; PUFA: polyunsaturated fatty acid; ND: not detected.

## References

[1] Mori T.A. (2017). Marine OMEGA-3 fatty acids in the prevention of cardiovascular disease. Fitoterapia.

[2] Simopoulos A.P. (2008). The importance of the omega-6/omega-3 fatty acid ratio in cardiovascular disease and other chronic diseases. Exp. Biol. Med..

[3] Lordan R., Tsoupras A., Zabetakis I. (2017). Phospholipids of animal and marine origin: Structure, function, and anti-inflammatory properties. Molecules.

[4] Nasopoulou C., Tsoupras A.B., Karantonis H.C., Demopoulos C.A., Zabetakis I. (2011). Fish polar lipids retard atherosclerosis in rabbits by down-regulating PAF biosynthesis and up-regulating PAF catabolism. Lipids Health Dis..

[5] Tsoupras A.B., Iatrou C., Frangia C., Demopoulos C.A. (2009). The implication of platelet-activating factor in cancer growth and metastasis: Potent beneficial role of PAF-inhibitors and antioxidants. Infect. Disord. Drug Targets.

[6] Tsoupras A.B., Chini M., Tsogas N., Fragopoulou E., Nomikos T., Lioni A., Mangafas N., Demopoulos C.A., Antonopoulou S., Lazanas M.C. (2008). Anti-platelet-activating factor effects of highly active antiretroviral therapy (HAART): A new insight in the drug therapy of HIV infection?. AIDS Res. Hum. Retrovir..

[7] Demopoulos C.A., Karantonis H.C., Antonopoulou S. (2003). Platelet-activating factor—A molecular link between atherosclerosis theories. Eur. J. Lipid Sci. Technol..

[8] Tsoupras A., Lordan R., Zabetakis I. (2018). Inflammation, not cholesterol, is a cause of chronic disease. Nutrients.

[9] Lordan R., Zabetakis I. (2017). Invited review: The anti-inflammatory properties of dairy lipids. J. Dairy Sci..

[10] Lordan R., Tsoupras A., Mitra B., Zabetakis I. (2018). Dairy fats and cardiovascular disease: Do we really need to be concerned?. Foods.

[11] Megson I.L., Whitfield P.D., Zabetakis I. (2016). Lipids and cardiovascular disease: Where does dietary intervention sit alongside statin therapy?. Food Funct..

[12] Zabetakis I. (2013). Food security and cardioprotection: The polar lipid link. J. Food Sci..

[13] Nasopoulou C., Karantonis H.C., Perrea D.N., Theocharis S.E., Iliopoulos D.G., Demopoulos C.A., Zabetakis I. (2010). In vivo anti-atherogenic properties of cultured gilthead sea bream (*Sparus aurata*) polar lipid extracts in hypercholesterolaemic rabbits. Food Chem..

[14] Sioriki E., Nasopoulou C., Demopoulos C.A., Zabetakis I. (2015). Comparison of sensory and cardioprotective properties of olive-pomace enriched and conventional gilthead sea bream (*Sparus aurata*): The effect of grilling. J. Aquat. Food Prod. Technol..

[15] Nasopoulou C., Smith T., Detopoulou M., Tsikrika C., Papaharisis L., Barkas D., Zabetakis I. (2014). Structural elucidation of olive pomace fed sea bass (*Dicentrarchus labrax*) polar lipids with cardioprotective activities. Food Chem..

[16] Nasopoulou C., Psani E., Sioriki E., Demopoulos C.A., Zabetakis I. (2013). Evaluation of sensory and in vitro cardio protective properties of sardine *(Sardina pilchardus*): The effect of grilling and brining. Food Nutr. Sci..

[17] Nasopoulou C., Nomikos T., Demopoulos C., Zabetakis I. (2007). Comparison of antiatherogenic properties of lipids obtained from wild and cultured sea bass (*Dicentrarchus labrax*) and gilthead sea bream (*Sparus aurata*). Food Chem..

[18] Panayiotou A., Samartzis D., Nomikos T., Fragopoulou E., Karantonis H.C., Demopoulos C.A., Zabetakis I. (2000). Lipid fractions with aggregatory and antiaggregatory activity toward platelets in fresh and fried cod (*Gadus morhua*):  Correlation with platelet-activating factor and atherogenesis. J. Agric. Food Chem..

[19] Rementzis J., Antonopoulou S., Argyropoulos D., Demopoulos C.A. (1996). Biologically active lipids from *S. scombrus*. Platelet-Activating Factor and Related Lipid Mediators 2.

[20] Fragopoulou E., Nomikos T., Tsantila N., Mitropoulou A., Zabetakis I., Demopoulos C.A. (2001). Biological activity of total lipids from red and white wine/must. J. Agric. Food Chem..

[21] Fragopoulou E., Antonopoulou S., Tsoupras A., Tsantila N., Grypioti A., Gribilas G., Gritzapi H., Konsta E., Skandalou E., Papadopoulou A. Antiatherogenic properties of red/white wine, musts, grape-skins, and yeast. Proceedings of the 45th International Conference on the Bioscience of Lipids, University of Ioannina.

[22] Megalemou K., Sioriki E., Lordan R., Dermiki M., Nasopoulou C., Zabetakis I. (2017). Evaluation of sensory and in vitro anti-thrombotic properties of traditional greek yogurts derived from different types of milk. Heliyon.

[23] Berdeaux O., Juaneda P., Martine L., Cabaret S., Bretillon L., Acar N. (2010). Identification and quantification of phosphatidylcholines containing very-long-chain polyunsaturated fatty acid in bovine and human retina using liquid chromatography/tandem mass spectrometry. J. Chromatogr. A.

[24] Zabetakis I. (2015). Marine Oils (From Sea to Pharmaceuticals).

[25] Raatz S.K., Johnson L.K., Rosenberger T.A., Picklo M.J. (2016). Twice weekly intake of farmed atlantic salmon (*Salmo salar*) positively influences lipoprotein concentration and particle size in overweight men and women. Nutr. Res..

[26] Zhang J., Wang C., Li L., Man Q., Song P., Meng L., Du Z.Y., Frøyland L. (2010). Inclusion of atlantic salmon in the chinese diet reduces cardiovascular disease risk markers in dyslipidemic adult men. Nutr. Res..

[27] Hagen I.V., Helland A., Bratlie M., Brokstad K.A., Rosenlund G., Sveier H., Mellgren G., Gudbrandsen O.A. (2016). High intake of fatty fish, but not of lean fish, affects serum concentrations of TAG and HDL-cholesterol in healthy, normal-weight adults: A randomised trial. Br. J. Nutr..

[28] Dalum A., Tangen R., Falk K., Hordvik I., Rosenlund G., Torstensen B., Koppang E.O. (2016). Coronary changes in the atlantic salmon *Salmo salar* L.: Characterization and impact of dietary fatty acid compositions. J. Fish Dis..

[29] Raatz S.K., Golovko M.Y., Brose S.A., Rosenberger T.A., Burr G.S., Wolters W.R., Picklo M.J. (2011). Baking reduces prostaglandin, resolvin, and hydroxy-fatty acid content of farm-raised atlantic salmon (*Salmo salar*). J. Agric. Food Chem..

[30] McLennan P.L., Owen A.J., Slee E.L., Theiss M.L. (2007). Myocardial function, ischaemia and n-3 polyunsaturated fatty acids: A membrane basis. Eur. J. Cardiovasc. Med..

[31] Rizos E.C., Ntzani E.E., Bika E., Kostapanos M.S., Elisaf M.S. (2012). Association between omega-3 fatty acid supplementation and risk of major cardiovascular disease events: A systematic review and meta-analysis. JAMA.

[32] Enns J.E., Yeganeh A., Zarychanski R., Abou-Setta A.M., Friesen C., Zahradka P., Taylor C.G. (2014). The impact of omega-3 polyunsaturated fatty acid supplementation on the incidence of cardiovascular events and complications in peripheral arterial disease: A systematic review and meta-analysis. BMC Cardiovasc. Disord..

[33] Kwak S., Myung S., Lee Y., Seo H. (2012). Efficacy of omega-3 fatty acid supplements (eicosapentaenoic acid and docosahexaenoic acid) in the secondary prevention of cardiovascular disease: A meta-analysis of randomized, double-blind, placebo-controlled trials. Arch. Intern. Med..

[34] Walz C.P., Barry A.R., Koshman S.L. (2016). Omega-3 polyunsaturated fatty acid supplementation in the prevention of cardiovascular disease. Can. Pharm. J..

[35] Chowdhury R., Stevens S., Gorman D., Pan A., Warnakula S., Chowdhury S., Ward H., Johnson L., Crowe F., Hu F.B. (2012). Association between fish consumption, long chain omega-3 fatty acids, and risk of cerebrovascular disease: Systematic review and meta-analysis. BMJ.

[36] Yanagita T., Nagao K. (2008). Functional lipids and the prevention of the metabolic syndrome. Asia Pac. J. Clin. Nutr..

[37] Küllenberg D., Taylor L.A., Schneider M., Massing U. (2012). Health effects of dietary phospholipids. Lipids Health Dis..

[38] Verouti S.N., Tsoupras A.B., Alevizopoulou F., Demopoulos C.A., Iatrou C. (2013). Paricalcitol effects on activities and metabolism of platelet activating factor and on inflammatory cytokines in hemodialysis patients. Int. J. Artif. Organs.

[39] Tsoupras A.B., Chini M., Mangafas N., Tsogas N., Stamatakis G., Tsantila N., Fragopoulou E., Antonopoulou S., Gargalianos P., Demopoulos C.A. (2012). Platelet-activating factor and its basic metabolic enzymes in blood of naive HIV-infected patients. Angiology.

[40] Ruiz-Lopez N., Stubhaug I., Ipharraguerre I., Rimbach G., Menoyo D. (2015). Positional distribution of fatty acids in triacylglycerols and phospholipids from fillets of atlantic salmon (*Salmo salar*) fed vegetable and fish oil blends. Mar. Drugs.

[41] Beppu F., Yasuda K., Okada A., Hirosaki Y., Okazaki M., Gotoh N. (2017). Comparison of the distribution of unsaturated fatty acids at the *sn*-2 position of phospholipids and triacylglycerols in marine fishes and mammals. J. Oleo Sci..

[42] Peng J., Larondelle Y., Pham D., Ackman R.G., Rollin X. (2003). Polyunsaturated fatty acid profiles of whole body phospholipids and triacylglycerols in anadromous and landlocked atlantic salmon (*Salmo salar* L.) fry. Comp. Biochem. Physiol. B Biochem. Mol. Biol..

[43] Simopoulos A. (2016). An increase in the omega-6/omega-3 fatty acid ratio increases the risk for obesity. Nutrients.

[44] Yeung E.H., Robledo C., Boghossian N., Zhang C., Mendola P. (2014). Developmental origins of cardiovascular disease. Curr. Epidemiol. Rep..

[45] O’Flaherty J.T., Tessner T., Greene D., Redman J.R., Wykle R.L. (1994). Comparison of 1-*O*-alkyl-, 1-*O*-alk-1′-enyl-, and 1-*O*-acyl-2-acetyl-*sn*-glycero-3-phosphoethanolamines and -3-phosphocholines as agonists of the platelet-activating factor family. Biochim. Biophys. Acta.

[46] Tsantila N., Karantonis H.C., Perrea D.N., Theocharis S.E., Iliopoulos D.G., Antonopoulou S., Demopoulos C.A. (2007). Antithrombotic and antiatherosclerotic properties of olive oil and olive pomace polar extracts in rabbits. Mediat. Inflamm..

[47] Hayashi H., Tanaka Y., Hibino H., Umeda Y., Kawamitsu H., Fujimoto H., Amakawa T. (1999). Beneficial effect of salmon roe phosphatidylcholine in chronic liver disease. Curr. Med. Res. Opin..

[48] Fukunaga K., Hossain Z., Takahashi K. (2008). Marine phosphatidylcholine suppresses 1,2-dimethylhydrazine-induced colon carcinogenesis in rats by inducing apoptosis. Nutr. Res..

[49] Hossain Z., Konishi M., Hosokawa M., Takahashi K. (2006). Effect of polyunsaturated fatty acid-enriched phosphatidylcholine and phosphatidylserine on butyrate-induced growth inhibition, differentiation and apoptosis in caco-2 cells. Cell Biochem. Funct..

[50] Bligh E.G., Dyer W.J. (1959). A rapid method of total lipid extraction and purification. Can. J. Biochem. Phys..

[51] Galanos D.S., Kapoulas V.M. (1962). Isolation of polar lipids from triglyceride mixtures. J. Lipid Res..

[52] Tsantila N., Tsoupras A.B., Fragopoulou E., Antonopoulou S., Iatrou C., Demopoulos C.A. (2011). In vitro and in vivo effects of statins on platelet-activating factor and its metabolism. Angiology.

[53] Nasopoulou C., Stamatakis G., Demopoulos C.A., Zabetakis I. (2011). Effects of olive pomace and olive pomace oil on growth performance, fatty acid composition and cardio protective properties of gilthead sea bream (*Sparus aurata*) and sea bass (*Dicentrarchus labrax*). Food Chem..

[54] Otero P., Saha S.K., Gushin J.M., Moane S., Barron J., Murray P. (2017). Identification of optimum fatty acid extraction methods for two different microalgae phaeodactylum tricornutum and haematococcus pluvialis for food and biodiesel applications. Anal. Bioanal. Chem..

[55] Qandil A. (2012). Prodrugs of nonsteroidal anti-inflammatory drugs (NSAIDS), more than meets the eye: A critical review. Int. J. Mol. Sci..

